# Outcome Analysis of Single Stage Total Knee Arthroplasty with Long Tibial Stem in Complex Primary Osteoarthritis with Tibia Stress Fracture

**DOI:** 10.5704/MOJ.2603.012

**Published:** 2026-03

**Authors:** S Singh, R Patel, SK Sonu, S Patel, SK Yadav, H Nagar

**Affiliations:** Department of Orthopaedics, Institute of Medical Sciences Banaras Hindu University, Varanasi, India

**Keywords:** osteoarthritis, stress fracture, total knee replacement, TKA with long tibial stem, proximal tibia stress fracture

## Abstract

**Introduction:**

The treatment of severe osteoarthritis of knee with proximal tibia stress fracture is daunting. There is no palpable treatment guideline for this condition. This study evaluates the functional and radiological outcomes of single staged long tibial stem total knee arthroplasty.

**Materials and methods:**

In this retrospective cohort, 10 patients were enrolled with severe osteoarthritis of knee with tibia stress fracture from 2020 to 2023. All patients were operated with posterior stabilising long tibial stem total knee arthroplasty. Pre-operative and post-operative functional and radiological assessments were evaluated.

**Results:**

The follow-up period ranged from 16 to 28 months, with a mean of 21 months. 15.2° varus was the average HKA angle (SD 3.68°). Knee range of motion increased from 106° (range 90 – 115°) to 127° (range 120 – 130°), while FFD improved from 16° (range 5 – 30°) to 1.5° (range 0 – 5°). Both the mean Knee Society score, and its functional score increased from 21.5 (range 5–35) to 78 (range 65–90) (p<0.001) and from 21.7 (range 4-42) to 89.3 (range 82-95) (p<0.001), respectively. All patients had fracture union during a follow-up period of three to six months (mean 3.90 months).

**Conclusion:**

Stress fracture in not uncommon in elderly osteoarthritic patients, long tibial stem total knee replacement provides safe and sublime result. Extension rod provides better restoration of alignment and facilitates fracture healing.

## Introduction

Stress fracture occurs due to repeated microtrauma on a bone over a long period of time^1^. When it occurs in a normal bone, it causes fatigue fracture, as in athletes, and when it occurs on abnormal underlying bone, it causes insufficiency fracture, as in elderly people^2^. It is commonly seen in patients with osteoporosis but can also be seen in patients with rheumatoid arthritis, hyperparathyroidism, and even after Total Joint Replacement surgery^3-8^.

Clinically, stress fracture presents insidious onset pain with no history of acute trauma. Early radiographs are not always helpful in diagnosis, but as the disease advances, periosteal bone reactions and horizontal or oblique patterns of sclerosis, a fracture line may be visible on radiograph films^9^. The management of patients with severe osteoarthritis (OA) with an ipsilateral stress fracture in the proximal tibia is complex and requires special attention.

Various treatment modalities are documented in the literature like treating the stress fracture alone conservatively on a cast but it require prolonged immobilisation and often result in joint stiffness and malalignment, Other surgical modalities includes open reduction internal fixation (ORIF) with a plate, or closed reduction internal fixation(CRIF) with an IM nail^10,11^, and then after healing of the fracture, the second stage of total joint replacement, or managing both the problems with single-stage total knee replacement with a long-stem tibial component which not only restores limb alignment but also helps in fracture union and enhances faster recovery in elderly patients^12^.

The purpose of this study was to retrospectively evaluate the functional and radiological outcomes of patients with severe osteoarthritis and an ipsilateral proximal tibia stress fracture treated with a single-stage long-stem total knee replacement.

## Materials and Methods

A retrospective cross-sectional study was conducted in patients of Kellgren and Lawrence (KL) Grade 3 and 4 osteoarthritis with proximal tibia stress fracture (Kaeding-Miller Grade 3, 4 and 5) from 2019 to 2023 after ethical approval (Declaration of Helsinki, 1964). The exclusion criteria were abnormal bone diseases like paget disease, hyperparathyroidism, metastasis, and traumatic fracture.

Fracture was diagnosed radiographically. Kellgren-Lawrence was used to classify knee osteoarthritis and Kaeding-Miller classification was used to classify stress fracture^13,14^. Metabolic disease work up like serum calcium levels, serum phosphate levels, Vitamin D levels, and parathyroid hormone levels was done.

**Fig. 1:**
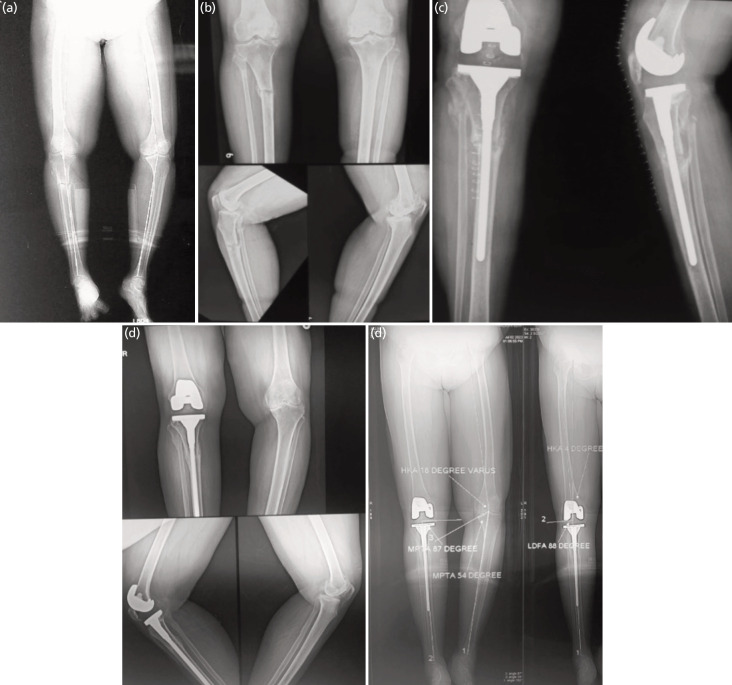
(a) Bilateral lower limb scanogram showing radiological parameters suggesting bilateral varus deformity of knee. (b) Standing antero-posterior and lateral radiograph showing severe osteoarthritis of knee with chronic stress fracture at junction of metaphysis and diaphysis of tibia. (c) Post total knee arthroplasty radiograph showing tibial stem crossing the tibial stress fracture site. (d) Two years post-operative follow-up radiographs showing complete union of tibia with no signs of loosening of implants and fractures. (e) Two years post-operative scanogram showing improved radiological parameters on operated side.

Surgical intervention was performed under combined epidural and spinal anaesthesia The tourniquet was inflated in the beginning and deflated once the implantation was done, and a standard midline skin approach was used. A medial parapatellar arthrotomy was performed, followed by bone cuts and soft tissue balancing. Serial reaming of the tibial canal was done with the hand reamer, and posterior stabilised prosthesis (Depuy, PFC sigma with extension rod) was used in all the patients. The length of the rod was determined pre-operatively by bypassing the fracture site on a radiograph and confirmed intra-operatively on fluoroscopy. The cementing of the tibial plate was done very carefully; care was taken to avoid cement intrusion into the canal and fracture site. Partial fibulectomy was done in case of stress fracture non-union (Kaeding-Miller Grade 5). We followed standard deep vein thrombosis (DVT) prophylaxis protocol^15-17^ and started injection Enoxaparin 0.4ml S/C 12–24 hours prior to surgery and then 0.6ml S/C per day at least for 2 weeks, followed by oral tablets of Ecospirin 150mg for 4-6 weeks. Post-operatively DVT pump was used in every patient. Patients were strictly advised non-weight bearing for two weeks, and once the sutures were out, we started mobilising them with partial weight bearing on a walker. After six weeks post-operatively, we started full weight-bearing walking with walker support when visible sign of callous formation appeared on radiographs. A regular follow-up was done at two weeks, six weeks, three months, six months, and yearly thereafter (Fig. 1).

Clinical assessment was assessed by fixed flexion deformity (FFD), knee range of motion (ROM), Knee Society score (KSS), Knee Society Functional score (KFSS)^18^ and Radiological assessment was done by full-length alignment radiographs, anteroposterior view, and lateral view to assess the union, medial proximal tibial angle (MPTA) (normal range 85-90°) and hip knee ankle angle (HKA) (normal range 1-1.5° varus)^19-21^.

For statistical analysis, IBM SPSS Version 21.0 for Windows Inc. used for all data analysis. Descriptive statistics were presented by frequency, percentage and mean (SD). Based on normality assumption, pre-operative and post-operative data comparison was done using Wilcoxon signed test. A p-value less than 0.05 is considered as statistically significant at 5% level of significance. Multiple bar diagram was used to represent two sets of inter-related data.

## Results

Over a span of four years, 10 patients were enrolled in our study. The mean age was 69.4 years ranging from 63 to 78 years. The cohort consisted of 7 females and 3 males giving a female is to male ratio 2.3:1.7 cases (70%) involved the right side, and 3 cases (30%) involved the left side. Seven patients had bilateral osteoarthritis of the knee joints; among them, five patients underwent total knee arthroplasty of the next knee after three months of previous Total knee Arthroplasty (TKA). The median follow-up was 20 months ranging from^16-28^ months (Table I).

**Table I:** Age of the patient, follow-up period and Union period of fracture.

	Mean	Median	SD	Minimum	Maximum
Age (years)	69.40	69.00	6.35	60.00	80.00
Follow-up (months)	21.00	20.00	3.92	16.00	28.00
Union (months)	3.90	4.00	0.99	3.00	6.00

**Fig. 2:**
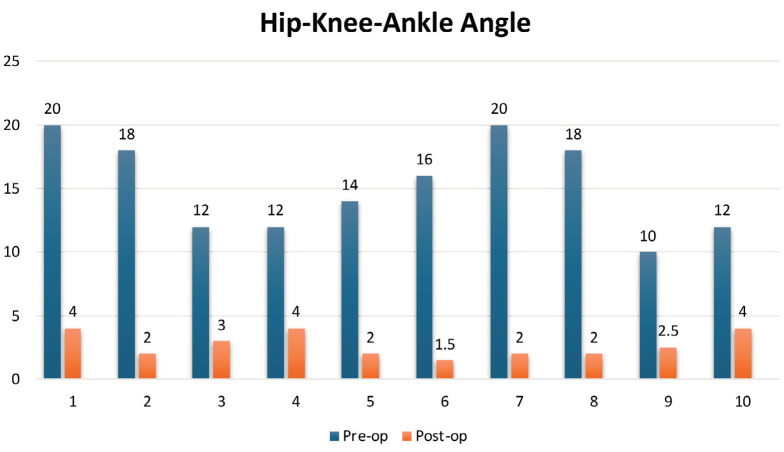
Hip knee ankle angle, pre- and post-operative.

**Fig. 3:**
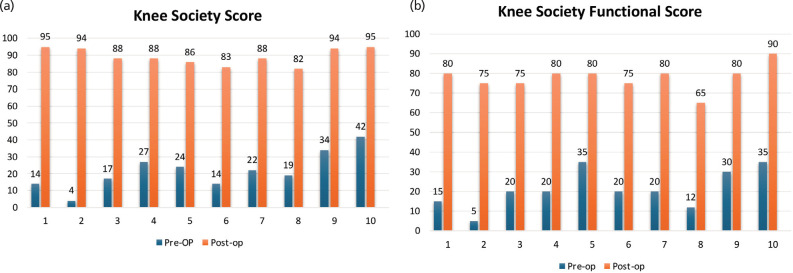
(a) Knee society score, pre- and post-operative. (b) Knee society functional score, pre- and post-operative.

In pre-operative assessment all patients had moderate varus (HKA 10 – 20° varus). The mean HKA angle was 15.2° varus (SD 3.68°). The mean MPTA angle was 68.7° (SD 8.64°). The mean FFD was 16° (range 5 – 30°), and the mean Knee range of motion was 106° (range 90 – 115°). Postoperatively, the mean HKA angle improved from 15.2° (range 10 – 20°) to 2.7° (range 1.5 – 4°). (Fig. 2) MPTA improved from 68.7° (range 55 – 79°) to 87.9° (range 85 – 89°). FFD was improved from 16° (range 5 – 30°) to 1.5° (range 0 – 5°), and Knee range of motion improved from 106° (range 90 – 115°) to 127° (range 120 – 130°) (Table II). The mean KSS improved from 21.7 (range 4 – 42) to 89.3 (range 82 – 95) (p<0.001) and the Mean KFSS improved from 21.5 (range 5 – 35) to 78 (range 65 – 90) (p<0.001) (Fig. 3). The fracture showed union within 3 to 6 months of follow-up (mean 3.90 months). In our study no patient showed any complications like infection, wound dehiscence, or implant failure.

**Table II:** Pre-operative and one-year post-operative follow-up, functional and radiological assessment.

	**Pre-operative (Mean±SD)**	**Post-operative (Mean±SD)**	**p value**
FFD	16±7.38	1.5±2.42	<0.001
KNEE ROM	106.5±8.52	127±4.22	<0.001
KSS	21.7±10.84	89.3±4.92	<0.001
KFSS	21.5±9.44	78±6.33	<0.001
MPTA	68.7±8.64	87.9±1.29	<0.001
HKA	15.2±3.68	2.7±0.98	<0.001

## Discussion

Stress fracture in elderly are mostly insufficiency fractures, the shaft of the tibia is the most common site^22^. The majority of them occur at the proximal half of the tibia^23^. Walking on abnormal bones causes repetitive stress in proximal part of tibia which increases coronal deformity like genu varus and valgus, causing shift of mechanical axis, which on walking further increases abnormal stress and loading on medial and lateral part of the proximal tibia, respectively. Insidious onset of pain, no history of trauma, lack of awareness and ignorance of the patient towards the additional pain in the knee, lack of suspicion, and not ordering full-length radiograph by the treating surgeons usually contribute to the delay or missed diagnosis of the stress fracture. Full-length radiographs are sufficient to make a diagnosis of stress fracture^9^, but in cases of suspicion, a bone scan and MRI can be an aid to diagnosis^24^. Pre-operative assessment of metabolic bone disease, serum levels of calcium phosphate, alkaline phosphatase, and parathyroid levels is encouraged.

There has been an ongoing debate in the management of severe OA with stress fracture. Some consider managing the stress fracture first, and total knee arthroplasty later on. Managing the stress fracture alone with a cast requires prolonged immobilisation, which eventually requires surgery because of the subsequently developed delayed union, malunion, pseudoarthrosis and malalignment which further increases joint stiffness and severity of arthritic symptoms^25,26^. Various Surgical modalities of stress fractures have been suggested like Intramedullary nailing or proximal tibia corrective osteotomy, plate fixation for stability with or without the use of a bone graft, and later a standard Total knee arthroplasty^10,11^.

Over a span of four years, we found that single staged TKA with long tibial stem in patients with severe OA and proximal tibia stress fracture is a sublime treatment modality for alignment and fracture union which is supported by numerous studies^9,27,28^.

**Fig. 4:**
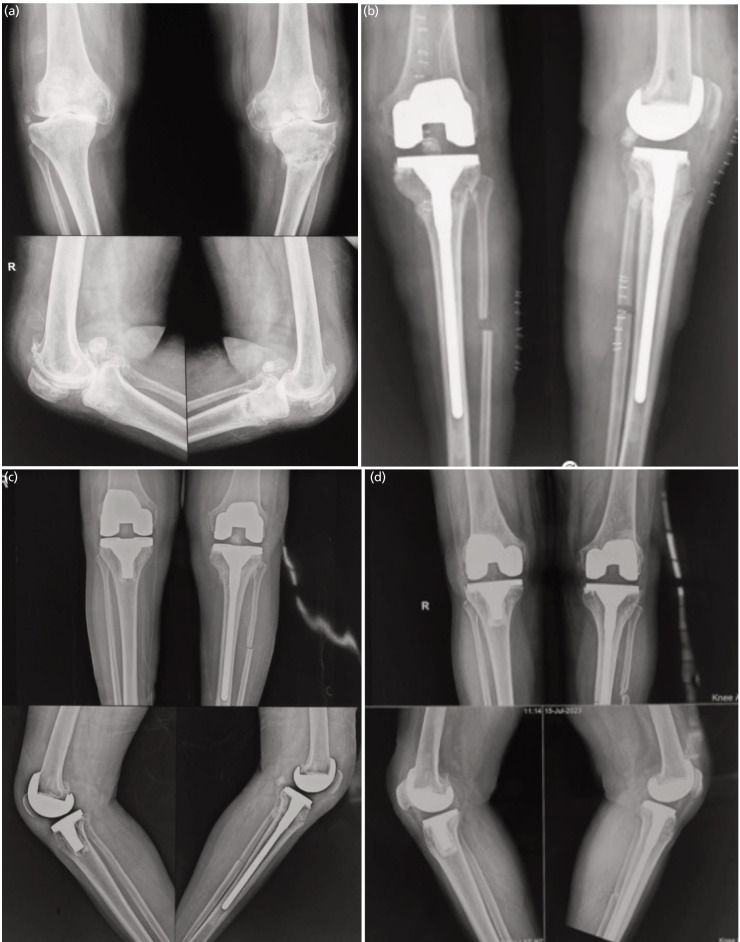
(a) Radiographs showing severe osteoarthritis of both knees with chronic stress fracture on left side with non-union. (b) Post TKA radiograph showing long tibial stem implant and Fibulectomy. (c) One-year follow-up Radiographs. (d) Two years follow-up radiographs showing complete union of tibia.

Extension rod on the tibial component provides alignment and internal splinting of the fracture by bypassing it. Soundarrajan *et al* also found that tibial stress fractures healed by four months post-long stem TKA^27^. Sawant *et al* achieved profound union with TKA with long stem in TKA valgus knees with severe osteoarthritis^28^ A critical insight we found in our study was that Partial fibulectomy increased the compressive strain of the tibia anteromedially and helped in closing the gap in Stress fracture non-union (Fig. 4).

There is a probability of periprosthetic fracture during or after the insertion of the tibial component with an extension rod. The keynote to highlight is that good pre-operative planning and angulation component correction before insertion of a tibial component with a long stem can avoid such complications which was in the line with the study proposed by Mittal *et al*^9^. To avoid a mismatch between the components it is recommended that the tibial stem and tibial base plate should be articulated by the same company supported by Pai S *et al*^29^. Some authors also use supplementary plate fixation with a long tibial stem^30^, but we didn’t require an additional plate or bone graft.

There were certain limitations in our study like small sample size, short follow-up and lack of control group.

## Conclusion

Single staged TKA is a safe and implicit treatment option in patients with severe osteoarthritis and an ipsilateral Proximal tibia stress fracture. It causes union of the stress fracture and corrects alignment at the fracture site and knee joint, hence, providing satisfactory functional and radiological outcomes.
